# Complete Genome Sequence of Methanofollis aquaemaris BCRC 16166^T^, Isolated from a Marine Aquaculture Fishpond

**DOI:** 10.1128/mra.00743-22

**Published:** 2022-09-12

**Authors:** Sheng-Chung Chen, Shu-Jung Lai, Yi-Ting You, Chao-Jen Shih, Yen-Chi Wu, Chih-Hung Wu, Ching-Hua Liao

**Affiliations:** a School of Chemistry and Chemical Engineering, Hubei Polytechnic University, Huangshi City, Hubei, People’s Republic of China; b School of Resources and Chemical Engineering, Sanming University, Sanming City, Fujian, People’s Republic of China; c Department of Life Sciences, National Chung Hsing University, Taichung City, Taiwan, Republic of China; d Graduate Institute of Biomedical Sciences, China Medical University, Taichung City, Taiwan, Republic of China; e Research Center for Cancer Biology, China Medical University, Taichung City, Taiwan, Republic of China; f Bioresource Collection and Research Center, Food Industry Research and Development Institute, Hsinchu, Taiwan, Republic of China; g College of Environment and Safety Engineering, Fuzhou University, Fuzhou City, Fujian, People’s Republic of China; Montana State University

## Abstract

The hydrogenotrophic methanogen Methanofollis aquaemaris BCRC 16166^T^ (= N2F9704^T^ = DSM 14661^T^) was isolated from a marine aquaculture fishpond near Wang-gong (Taiwan, Republic of China). The genome of strain BCRC 16166^T^ was selected for sequencing in order to provide further information about the species delineation and its infected virus.

## ANNOUNCEMENT

Strains in the genus *Methanofollis* are hydrogenotrophic, and the type species is Methanofollis tationis (1, 2). To date (July 2022), a total of six *Methanofollis* species have been characterized and validly described, namely, Methanofollis tationis (2, 3), Methanofollis liminatans (3, 4), Methanofollis aquaemaris (5), Methanofollis formosanus (6), Methanofollis ethanolicus (7), and Methanofollis fontis (8). These isolates are widespread in various anaerobic environments, such as a solfataric field, a wastewater reactor, an aquaculture fishpond, a lotus field, and cold seep sediment (2, 4–8). Interestingly, the cells of M. aquaemaris were infected with novel coccus-shaped, enveloped viruses with a diameter of 200 nm (5). To date, over 100 archaeal viruses have been discovered, and most are related to thermophilic *Crenarchaea* and extreme halophilic *Euryarchaea* (9, 10). However, only a few methanoarchaeal viruses have been reported in the past 3 decades (11–17). Here, we report the complete genome of M. aquaemaris BCRC 16166^T^, which provides insight to further understand the interaction between archaeal virus and host, as well as microbial adaptation to various environments.

Strain BCRC 16166^T^ was obtained from the Bioresource Collection and Research Center (Taiwan, Republic of China), grown in anaerobic MB/W medium with 100 mM sodium formate and 5 mM sodium acetate, and incubated at 37°C, according to the methods described previously (6, 18, 19). Genomic DNA of strain BCRC 16166^T^ was isolated and purified using a modification of the methods described by Johnson (20) and Jarrell et al. (21). Briefly, cells from 0.5 L of culture were lysed with sodium dodecyl sulfate (SDS) (1% [wt/vol]). After phenol-chloroform extraction and isopropanol precipitation, the quantity and quality of extracted DNA samples were examined with a UV-visible spectrophotometer.

The genome of strain BCRC 16166^T^ was sequenced at the Genomics BioSci & Tech Co., Ltd. (Taiwan, Republic of China), using the MiSeq platform (Illumina). Genomic DNA was sheared randomly, and a paired-end DNA library of 300 bp was constructed by using the TruSeq Nano DNA high-throughput (HT) library preparation kit and TruSeq DNA with 96 combinatorial dual (CD) indexes (Illumina). The constructed library was sequenced using the MiSeq reagent kit v3 (600 cycles), and 6,956,336 reads were generated. All generated reads were quality trimmed to obtain high-quality reads using Trimmomatic v0.36 (22). These reads were *de novo* assembled by SPAdes v3.10.1 (23), and the quality of the assembled genome was evaluated by QUAST v4.5 (24). The sequencing protocol generated ~630× mean coverage of the genome. The assembly generated a single large contig of 2,746,416 bp, which was circularized by aligning the two ends of the contig sequences and deleting overlapping sequences at one end. Genes of the genome were identified using the National Center for Biotechnology Information (NCBI) Prokaryotic Genome Annotation Pipeline (PGAP) v4.7 (http://www.ncbi.nlm.nih.gov) (25). Default parameters were used for all software unless otherwise specified.

The complete genome of strain BCRC 16166^T^ had a total size of 2,746,416 bp and a GC content of 59.55%. The genome was predicted to harbor 2,581 genes. Two clustered regularly interspaced short palindromic repeats (CRISPRs) with a high evidence level were found in the genome by using CRISPRCasFinder (26).

### Data availability.

This whole-genome shotgun project has been deposited in GenBank under the accession number CP036172. The version described in this paper is the first version. The BioProject accession number is PRJNA269558. The raw sequence reads have been deposited in the Sequence Read Archive (SRA) under accession number SRR18077124.
